# Effects of Dietary Bopu Powder Supplementation on Serum Antioxidant Capacity, Egg Quality, and Intestinal Microbiota of Laying Hens

**DOI:** 10.3389/fphys.2022.902784

**Published:** 2022-07-22

**Authors:** Hua Liu, Qian Lin, Xiubin Liu, Peng Huang, Zihui Yang, Manhu Cao, Mengting Liu, Xinyao Li, Jianguo Zeng, Jianhua He

**Affiliations:** ^1^ College of Animal Science and Technology, Hunan Agricultural University, Changsha, China; ^2^ Hunan Key Laboratory of Traditional Chinese Veterinary Medicine, Hunan Agricultural University, Changsha, China; ^3^ Institute of Bast Fiber Crops, Chinese Academy of Agricultural Sciences, Changsha, China; ^4^ College of Veterinary Medicine, Hunan Agricultural University, Changsha, China

**Keywords:** Bopu powder, antioxidant capacity, egg quality, gut microbiota, laying hens

## Abstract

The purpose of this study was to investigate the effects of dietary Bopu powder supplementation on the serum antioxidant capacity, serum biochemical indices, egg quality, and intestinal microbiota. Six hundred and forty-eight 33-week-old Lohmann Brown commercial laying hens were randomly allocated into six groups and fed a basal diet supplemented with 0, 25, 50, 100, 200, and 400 mg/kg Bopu powder for 8 weeks, denoted BP0, BP25, BP50, BP100, BP200, and BP400, respectively. The results showed that dietary Bopu powder supplementation reduced serum cholesterol concentrations (linear, *p* < 0.01) while increasing serum globulin and albumin concentrations (linear, *p* < 0.05). Furthermore, the BP50 and BP100 groups had greater serum catalase and glutathione peroxidase activity (*p* < 0.05). The egg Haugh Units were considerably higher in BP25 and BP50 (*p* < 0.05), and eggshell thickness was higher in BP25, BP200, and BP400 (*p* < 0.05) when compared to BP0. Dietary treatment with Bopu powder at doses ranging from 25–100 mg/kg improved glutathione peroxidase and catalase activities while decreasing malondialdehyde concentrations in the yolk (*p* < 0.05). The addition of Bopu powder increased the diversity of microbiota and the relative abundance of Bacteroidota in the gut. For instance, dietary Bopu powder supplementation of 25–50 mg/kg significantly raised the relative abundance of *Enterococcus, Bacteroides*, and *Fusobacterium* in the foregut. Supplementing the diet with 50–100 mg/kg of Bopu powder improved the relative abundance of *Lactobacillus* in the hindgut. In conclusion, dietary Bopu powder supplementation enhanced the abundance of beneficial bacteria in the foregut of laying hens and improved egg quality and antioxidant capacity. Furthermore, in the laying hen diet, the optimal dosage of Bopu powder additive was 25–50 mg/kg.

## Introduction

Eggs are one of the most cost-effective sources of high biological value and well-balanced protein. The nutritional value and customer preferences are influenced by the eggshell and internal quality, which is crucial to the egg industry’s economic viability (Roberts, 2004). Antibiotic growth promoters (AGPs) are commonly used in food-animal feeds in modern animal husbandry to boost production performance and protect animals from diseases (Castanon, 2007). Although utilizing AGPs in laying hen diet increased laying production and egg quality, it also increased antibiotic residues in eggs and the potential for antibiotic-resistant strains of bacteria to cause health problems in humans. Thus, it is necessary to investigate alternative ways for improving laying performance, egg quality, and preventing laying hen diseases, to ensure healthy and sustainable laying hen production.

Natural plant (Chinese herbal medicine) extracts are known for their multi-functionality, no known bacteria resistance, and low toxicity. Recent studies indicate that natural plant extracts have positive effects on the laying performance and egg quality of laying hens (Abdelli et al., 2021; Righi et al., 2021). Natural antioxidants in the diet, such as tea polyphenols, may help to lay hens increase their performance, albumen quality, magnum shape, antioxidant status, and egg antioxidant capacity (Wang et al., 2018; Zhou et al., 2021). Wang et al. (2019) discovered that dietary tea polyphenol supplementation could mitigate the negative effects of high molybdenum exposure on performance, egg quality, and antioxidant status in laying hens. Also, dietary tea polyphenol supplementation differentially enriched microbial compositions in the cecum enhanced the enrichment of *Bacilli*, *Lactobacillates*, *Lactobacillus*, and *Lactobacillus gasseri*, and indicated that dietary tea polyphenols maintain eubiosis of the cecum microbiota in molybdenum-challenged layers (Wang et al., 2019).

Intestinal microbiota plays a vital role in maintaining gut health and influences the overall performance of laying hens. Gut microbiota is actively involved in the development of the immune system, can confer protection from pathogen infection *via* competitive exclusion and production of antimicrobial compounds, supplies micronutrients, amino acids, and short-chain fatty acids, and influences the development of intestinal epithelium (Khan et al., 2020). Throughout the laying hen production cycle, the gut microbiota composition is altered with age in distinct ways (Joat et al., 2021; Sun et al., 2021). Understanding the baseline and evolution of gut microbiota in laying hens throughout the course of their lives was crucial to obtaining optimal performance and gut health. According to new research, supplementing natural plant extracts enhances laying hen performance and egg quality while also modifying the gut microbiome (Kim et al., 2018; Abad et al., 2020; Dilawar et al., 2021).

*Macleaya cordata* (Chinese name “Bo-luo-hui”), also known as plume poppy, is a perennial traditional medicinal herb of the Papaveraceae family that is widely distributed in southern China. Its main active compounds are benzophenanthridine alkaloids (sanguinarine and chelerythrine) and protopine alkaloids (protopine and allocryptopine). Our previous studies showed that sanguinarine extracted from *Macleaya cordata* modulated the gut microbiome and intestinal morphology to enhance growth performance in broilers (Liu et al., 2020). Compounds comprising sanguinarine and chelerythrine derived from *Macleaya cordata* were recognized as feed additives in the European Union in 2005, and are widely utilized in poultry and livestock production to replace antibiotic growth promoters. In 2019, compounds containing protopine and allocryptopine isolated from *Macleaya cordata* were registered as veterinary medications in China as Bopu powder (Veterinary Drug No. 180415374), which can be used to treat chicken diarrhea caused by *E. coli*.

Propine and allocryptopine have a wide range of therapeutic biological actions, including anti-inflammatory, antibacterial, antiviral, liver repair, and neuroprotective properties (Vacek et al., 2010; Huang et al., 2021). However, there is limited research in the scientific literature on the effects of protopine and allotytopine on the health and egg quality of laying hens. As a result, the purpose of this study was to assess the effects of dietary supplementation with various amounts of Bopu powder on laying performance, egg quality, serum antioxidant capacity, and gut microbiota in laying hens.

## Materials and Methods

### Birds, Diets, and Management

Six hundred and forty-eight 33-week-old Lohmann laying hens with an initial egg production of 89.97% ± 6.05% were randomly and equally distributed among the six dietary treatments. Six experimental diets were formulated based on corn and soybean meal supplemented with 0, 25, 50, 100, 200, and 400 mg/kg Bopu powder, expressed as BP0, BP25, BP50, BP100, BP200, and BP400, respectively. At the expense of corn, Bopu powder was added to the basal diet (Table 1). The Bopu powder consisted of 1% protopine, 0.5% allotypotopine, and 98.5% starch, was produced by the Micolta Bioresource Company Ltd. (Changsha, 410331, PR China). Each treatment had 6 replicates with 18 hens each. Replicates were equally distributed into upper, middle, and lower cage levels to minimize the replicate level effect. Three hens were housed in a 45-by-45-by-45-cm cage, with six surrounding galvanized steel cages (two cages on each floor) serving as a replicate. Ambient temperature and humidity in the laying hen barn were maintained at 23 ± 2°C and 50 to approximately 65%, respectively. The photoperiod was set to 16L:8D throughout the study. All hens were given free access to feed and water. All of the birds were fed a basal diet for 2 weeks prior to the feeding trial (33 weeks of age). The experiment lasted 8 weeks (from 35 to 42 weeks of age). During the study, the animals were housed and handled according to the Lohmann Brown Laying Hens Management Guide’s guidelines.

**TABLE 1:** Composition and nutrient levels of basal diets (air-dry basis, %).

Items	Diets
Ingredients, %	
Corn	55.45
Soybean meal	29.8
Soybean Oil	1.6
Wheat bran	1.2
Limestone	8.45
CaHPO4·2H2O	1.2
NaCl	0.3
Premix^a^	2
Total	100.00
Nutrient content^b^	
Metabolic energy, MJ/kg	11.5
Crude protein, %	17.00
Crude fiber, %	3.14
Ca, %	3.50
Total phosphorus, %	0.55
Available phosphorus, %	0.33
Lys, %	0.95
Met, %	0.36
Met + Cys, %	0.65

a The premix provided the following (per kilogram of complete diet) micronutrients: VA, 6,000 IU, VD_3_ 2,500 IU, VE, 25 mg, VK_3_ 2.25 mg, VB_1_ 1.8 mg, VB_2_ 7 mg, VB_6_ 4 mg, VB_12_ 0.2 mg, *D*-pantothenic acid 12 mg, nicotinic acid 35 mg, biotin 0.14 mg, folic acid 0.8 mg, Cu (as copper sulfate) 11 mg, Zn (as zinc sulfate) 70 mg, Fe (as ferrous sulfate) 60 mg, Mn (as manganese sulfate) 115 mg, Se (as sodium selenite) 0.30 mg, and I (as potassium iodide) 0.4 mg.

b Nutrient levels are calculated values.

### Laying Performance and Sampling

The egg production and egg mass were recorded daily. The feed intake of each replicate was recorded weekly. The feed conversion ratio was calculated as the ratio of total feed consumed to total egg mass-produced. Egg production was expressed as an average daily production. At the end of the trial, a total of 36 hens (6 replicates/treatment, 1 hen/replicate) were randomly collected from each treatment and humanely slaughtered after a 12-h fast (water offered *ad libitum*) to collect foregut contents and hindgut contents. The collected intestinal contents were frozen immediately in liquid nitrogen, and then stored at −80°C for subsequent analyses. Before slaughter, blood was collected from the wing vein and centrifuged at 3,000 X g for 10 min to separate the serum, and then frozen at −20°C for further analysis.

### Egg Quality and Antioxidant Capacity of Yolk

Eggs (six replicates/treatment, eight eggs/replicate) were collected on the final day of the experiment to measure egg quality. Egg yolk color, albumen height, and Haugh unit were evaluated using an egg multitester (EMT-7300, Robotmation Co. Ltd., Tokyo, Japan). Eggshell breaking strength was evaluated using an eggshell force gauge model II (Robotmation Co. Ltd., Tokyo, Japan). Eggshell thickness was measured at the large end, equatorial region, and small end using an eggshell thickness gauge (Robotmation Co., Ltd., Tokyo, Japan). Eggshell ratio was calculated as eggshell weight/egg weight ⅹ100. The egg shape index was calculated as the length of the egg divided by its width. Total antioxidant capacity (T-TAOC), total superoxide dismutase (T-SOD) activity, glutathione peroxidase (GSH-Px) activity, catalase (CAT) activity, and malondialdehyde (MDA) content in yolk were determined by assay kits (Nanjing Jiancheng Bioengineering Institute, China) according to the manufacturer’s instructions.

### Serum Biochemical Parameters and Antioxidant Enzyme Activity

Serum total protein levels, albumin, globulin, total cholesterol (CHO), triglyceride (TG), urea, glucose (GLU), urea acid (UA), Ca, phosphorus (P), alkaline phosphatase (ALP), aspartate aminotransferase (AST), alanine aminotransferase (ALT), GSH-Px, superoxide dismutase (SOD), T-AOC, and MDA were assayed with commercial assay kits (Nanjing Jiancheng Bioengineering Institute, Nanjing, China) according to the manufacturer’s guidelines.

### Intestinal Microflora DNA Extraction, Library Preparation, and Sequencing

Total microbial genomic DNA was extracted from intestinal contents using the E. Z.N.A.^®^ soil DNA Kit (Omega Bio-tek, Norcross, GA, United States) according to the manufacturer’s instructions. The quality and concentration of DNA were determined using 1.0% agarose gel electrophoresis and a NanoDrop^®^ ND-2000 spectrophotometer (Thermo Scientific Inc., United States), which was then stored at −80°C until further use. The V3-V4 hypervariable region of the bacterial 16S rRNA gene was amplified with primer pairs 338F (5′-ACT​CCT​ACG​GGA​GGC​AGC​AG-3′) and 806R (5′-GGACTACHVGGGTWTCTAAT-3′) by an ABI GeneAmp^®^ 9700 PCR thermocycler (ABI, CA, United States). The PCR reaction mixture includes 4 μl 5 × Fast Pfu buffer, 2 μl 2.5 mM dNTPs, 0.8 μl each primer (5 μM), 0.4 μl Fast Pfu polymerase, 10 ng of template DNA, and ddH_2_O to a final volume of 20 µl. The cycling conditions for PCR amplification were as follows: initial denaturation at 95°C for 3 min, followed by 27 cycles of denaturing at 95°C for 30 s, annealing at 55°C for 30 s and extension at 72°Cfor 45 s, and single extension at 72°C for 10 min, and end at 4°C. All samples were amplified in triplicate. The PCR product was extracted from a 2% agarose gel and purified using the AxyPrep DNA Gel Extraction Kit (Axygen Biosciences, Union City, CA, United States) according to the manufacturer’s instructions and quantified using the Quantus™ Fluorometer (Promega, Madison, WA, United States). Purified amplicons were pooled in equimolar amounts and paired-end sequenced on an Illumina MiSeq PE300 platform/NovaSeq PE250 platform (Illumina, San Diego, United States) according to the standard protocols by Majorbio Bio-Pharm Technology Co. Ltd. (Shanghai, China). The raw sequencing reads were deposited into the NCBI Sequence Read Archive (SRA), PRJNA837752. After demultiplexing, the resulting sequences were quality filtered with fastp (0.19.6) and merged with FLASH (v1.2.11) as described previously by Chen et al. (2018) and Magoc and Salzberg (2011). Then the high-quality sequences were denoised using the DADA2 plugin in the Qiime2 (version 2020.2) as described previously by Callahan et al. (2016) and Bolyen et al. (2019) pipeline with recommended parameters, which obtains single-nucleotide resolution based on error profiles within samples. DADA2 denoised sequences are usually called amplicon sequence variants (ASVs).

### Statistical Analysis

Firstly, all the data were recorded and sorted in Excel. Then, data analysis of performance, egg quality, serum indices, and antioxidant capacity was performed using the IBM SPSS Statistics 22 statistical package (SPSS Inc., Chicago, IL, United States). The normality of the data was initially tested using the Shapiro–Wilk test. The data were then analyzed using one-way ANOVA and Orthogonal Polynomial Contrasts to determine linear and quadratic responses to different levels of Bopu powder. When the ANOVA showed statistical significance, Duncan’s multiple range test was conducted. Differences were considered statistically significant at *p* < 0.05. The *p* values between 0.05 and 0.10 were considered a trend. Data were expressed as the mean and pooled SEM.

Bioinformatic analysis of the gut microbiota was carried out using the Majorbio Cloud platform (https://cloud.majorbio.com). Based on the ASVs information, rarefaction curves and alpha diversity indices including observed ASVs, Chao1 richness, ace index, Shannon index, and Simpson index were calculated with Mothur v1.30.1 (Schloss et al., 2009). Similarity among the microbial communities in different samples was determined by principal coordinate analysis (PCoA) based on Bray-Curtis dissimilarity using the Vegan v2.5-3 package. The linear discriminant analysis (LDA) effect size (LEfSe) (http://huttenhower.sph.harvard.edu/LEfSe) was performed to identify the significantly abundant taxa (phylum to genus) of bacteria among the different groups (LDA score >2, *p* < 0.05) as described previously by Segata et al. (2011).

## Results

### Production Performance

The effect of dietary Bopu powder supplementation on the performance of laying hens was presented in Table 2. No mortality was found during the 8-week experimental period. The dietary Bopu powder supplementation had no significant effects on egg production, average egg weight, average daily feed intake, or feed conversion ratio.

**TABLE 2:** Effect of dietary Bopu powder supplementation on the performance of laying hen.

Item		Bopu powder, mg/kg						SEM	*p* value		
		0	25	50	100	200	400		ANOVA	Linear	Quadratic
Egg production, %	Week 1 to 4	89.25	89.88	90.04	90.59	90.48	91.11	0.627	0.977	0.373	0.676
	Week 5 to 8	89.66	89.00	89.93	88.69	89.90	88.41	0.758	0.989	0.759	0.936
	Week 1 to 8	89.46	89.44	89.99	89.64	90.19	89.76	0.643	0.999	0.802	0.959
Average egg mass, g/hen/day	Week 1 to 4	51.88	53.20	52.82	52.80	53.36	53.45	0.400	0.906	0.313	0.587
	Week 5 to 8	54.54	54.94	55.66	54.76	55.26	54.23	0.461	0.967	0.857	0.479
	Week 1 to 8	53.20	54.10	54.22	53.78	54.28	53.85	0.387	0.979	0.688	0.783
Average daily feed intake, g/hen/day	Week 1 to 4	115.14	115.00	115.10	114.76	115.01	114.87	0.057	0.714	0.173	0.371
	Week 5 to 8	116.02	116.04	116.00	116.02	116.08	116.03	0.015	0.943	0.478	0.777
	Week 1 to 8	115.58	115.52	115.56	115.38	115.57	115.45	0.031	0.714	0.308	0.563
FCR, g/g	Week 1 to 4	2.23	2.17	2.18	2.19	2.16	2.16	0.017	0.840	0.266	0.497
	Week 5 to 8	2.13	2.12	2.09	2.13	2.11	2.15	0.018	0.971	0.795	0.751
	Week 1 to 8	2.18	2.14	2.14	2.16	2.13	2.15	0.016	0.966	0.662	0.740

FCR, feed conversion ratio.

### Serum Biochemical Parameter Indices

The effect of dietary Bopu powder supplementation on serum biochemical parameters indices of laying hens was presented in Table 3. Serum CHO concentrations decreased (linear*, p < 0.01*) with increasing Bopu powder supplementation, whereas serum GLB and ALB concentrations increased (linear, *p < 0.05*) in laying hens. Dietary supplementation with Bopu powder had no influence on serum GLU, TG, UA, ALT, or AST levels.

**TABLE 3:** Effect of dietary Bopu powder supplementation on serum biochemical parameters indices of laying hens.

Item	Bopu powder, mg/kg						SEM	*p* value		
	0	25	50	100	200	400		ANOVA	Linear	Quadratic
GLU, mmol/L	10.00	10.21	10.90	10.80	11.02	11.07	0.156	0.214	0.052	0.053
TG, mmol/L	24.31	23.89	25.82	24.76	22.05	22.17	0.637	0.530	0.117	0.298
CHO, mmol/L	5.42^b^	5.58^b^	5.41^b^	5.36^b^	5.01^a^	4.90^a^	0.064	<0.001	<0.001	<0.001
UA, umol/L	0.223	0.210	0.220	0.213	0.277	0.270	0.014	0.689	0.128	0.300
GLB, g/L	67.27^b^	77.99^a^	71.68^ab^	67.4^b^	70.44^ab^	75.13^ab^	2.673	0.045	0.017	0.337
ALB, g/L	14.91^b^	14.13^b^	14.20^b^	14.04^b^	15.48^a^	15.48^a^	0.195	0.038	0.021	0.077
TP, g/L	82.18^b^	92.12^a^	85.88^ab^	81.44^b^	85.92^ab^	90.61^a^	3.430	0.023	0.653	0.464
ALT, U/L	5.276	4.698	5.899	5.99	5.565	4.64	0.233	0.431	0.416	0.238
AST, U/L	210.43	234.47	223.27	243.33	187.67	197.83	7.22	0.180	0.112	0.293

^a,b^Means in the same row with different superscript letters indicate differences (*p* < 0.05).

GLU, glucose; TG, triglyceride; CHO, cholesterol; UA, uric acid; GLB, globulin; ALB, albumin; TP, total protein; ALT, glutamic-pyruvic transaminase; AST, glutamic-oxaloacetic transaminase.

### Antioxidant Capacity of Serum

Table 4 shows that serum GSH-Px activity increased significantly in the Bopu powder supplemented groups (50–100 mg/kg) compared to the BP0 group (*p* < 0.05). Serum CAT activity in laying hens was increased (linear, *p* < 0.05) when Bopu powder supplementation increased.

**TABLE 4:** Effect of dietary Bopu powder supplementation on serum antioxidant capacity of laying hens.

Item	Bopu powder, mg/kg						SEM	*p* value		
	0	25	50	100	200	400		ANOVA	Linear	Quadratic
T-AOC, mmol/L	1.10	1.21	1.20	1.40	1.20	1.33	0.040	0.362	0.238	0.407
T-SOD, U/ml	722.32	682.78	703.92	712.93	736.85	649.18	12.94	0.475	0.238	0.172
GSH-Px, U/ml	175.86^a^	219.36 ^ab^	240.00^b^	257.49^b^	227.52 ^ab^	256.12^b^	88.17	0.042	0.062	0.086
GSH, umol/L	40.11	44.23	41.80	40.71	44.43	45.51	1.034	0.634	0.170	0.403
CAT, U/ml	3.8^b^	8.0^a^	7.9^a^	7.6^a^	8.8^a^	8.1^a^	0.110	0.001	0.032	0.585
MAD, nmol/ml	8.66	8.33	7.50	8.26	8.15	6.79	0.804	0.993	0.557	0.837

^a,b^Means in the same row with different superscript letters indicate differences (*p* < 0.05).

T-AOC, total antioxidative capacity; T-SOD, total superoxide dismutase; GSH-Px, glutathione peroxidase; GSH, glutathione; CAT, catalase; MAD, malondialdehyde.

### Egg Quality and Antioxidant Capacity of the Yolk

As shown in Table 5, the egg Haugh units and eggshell thickness were significantly affected by dietary Bopu powder supplementation (*p* < 0.05). Compared with the BP0 group, BP25 and BP50 groups significantly increased egg Haugh units (*p* < 0.05), and BP25, BP200, and BP400 groups significantly enhanced eggshell thickness (*p* < 0.05). The effects of dietary Bopu powder supplementation on antioxidant enzyme activities in the yolk were presented in Table 6. The Bopu powder supplementation groups (25–100 mg/kg) had significantly higher yolk GSH-Px activity than the BP0 group (*p* < 0.05). Furthermore, the BP25 and BP50 groups exhibited significantly higher CAT activity in the yolk (*p* < 0.05) than the BP0 group; the MDA concentration was greatly lowered in the BP25 group (*p* < 0.05) but significantly increased in the BP200 group (*p* < 0.05).

**TABLE 5:** Effect of dietary Bopu powder supplementation on egg quality of laying hens (8 weeks).

Item	Bopu powder, mg/kg						SEM	*p* value		
	0	25	50	100	200	400		ANOVA	Linear	Quadratic
Egg shape index	1.35	1.35	1.34	1.36	1.32	1.34	0.005	0.499	0.181	0.285
Eggshell strength, kgf	4.60	4.73	4.52	4.30	4.56	4.54	0.064	0.574	0.769	0.645
Egg weight, g	60.78	61.60	62.22	59.96	60.81	61.58	0.257	0.085	0.432	0.812
Haugh units	58.15^b^	66.42^a^	65.08^a^	64.59^ab^	58.76^b^	58.40^b^	1.039	0.046	0.065	0.172
Yolk color score	4.10	3.90	4.00	3.83	4.00	3.90	0.146	0.981	0.777	0.933
Yolk width, mm	39.65	40.03	38.93	40.36	39.33	40.48	0.263	0.534	0.367	0.560
Yolk height, mm	16.55	16.39	16.32	16.30	16.16	16.42	0.113	0.960	0.841	0.616
Yolk weight, %	27.19	28.20	28.53	27.78	27.47	27.39	0.214	0.431	0.365	0.630
Shell thickness, mm	0.335^b^	0.352^a^	0.346^ab^	0.332^b^	0.351^a^	0.351^a^	0.002	0.001	0.080	0.191
Shell weigh, %	9.36	9.47	9.44	9.79	9.80	9.54	0.080	0.513	0.416	0.150

^a,b^Means in the same row with different superscript letters indicate differences (*p* < 0.05).

**TABLE 6:** Effect of dietary Bopu powder supplementation on the antioxidant capacity of yolk.

Item	Bopu powder, mg/kg						SEM	*p* value		
	0	25	50	100	200	400		ANOVA	Linear	Quadratic
T-AOC, U/ml	15.57	16.38	13.09	12.24	13.55	13.55	1.014	0.866	0.010	0.064
T-SOD, U/ml	425.00	397.58	451.00	372.08	427.58	350.17	23.248	0.135	0.235	0.552
GSH-Px, U/ml	207.19^b^	490.13^a^	690.56^a^	390.77^a^	215.45^b^	345.92^ab^	50.955	0.034	0.130	0.268
CAT, U/ml	65.15^b^	73.55^a^	72.85^a^	67.60^ab^	69.65^ab^	61.74^b^	3.358	0.001	0.685	0.207
MDA, nmol/ml	204.25^b^	144.80^a^	170.30^ab^	177.14^ab^	294.16^c^	212.00^b^	11.810	0.001	0.004	0.033

^a,b,c^Means in the same row with different superscript letters indicate differences (*p* < 0.05).

T-AOC, total antioxidative capacity; T-SOD, total superoxide dismutase; GSH-Px, glutathione peroxidase; GSH, glutathione; CAT, catalase; MAD, malondial.

### Intestinal Microbiota

**FIGURE 1:**
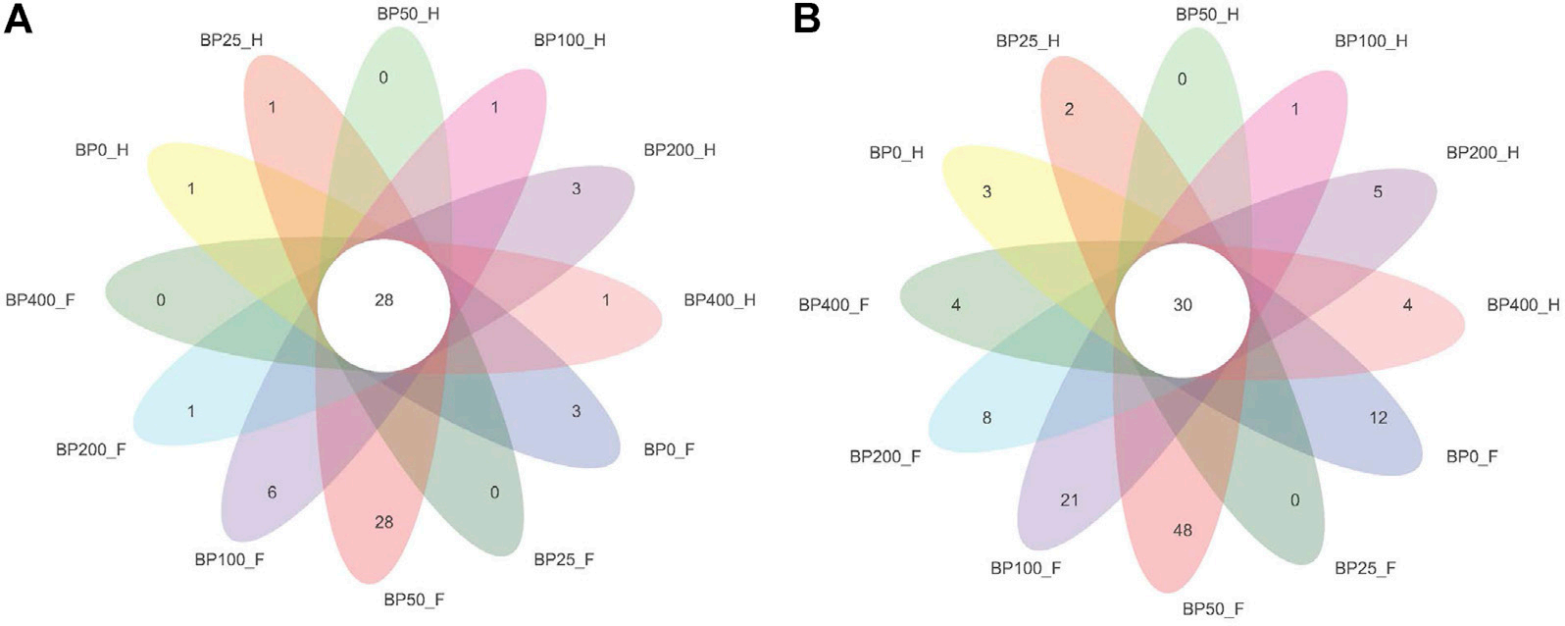
**(A)** Venn diagram of intestinal microbiota at family level. **(B)** Venn diagram of intestinal microbiota at genus level.

**FIGURE 2:**
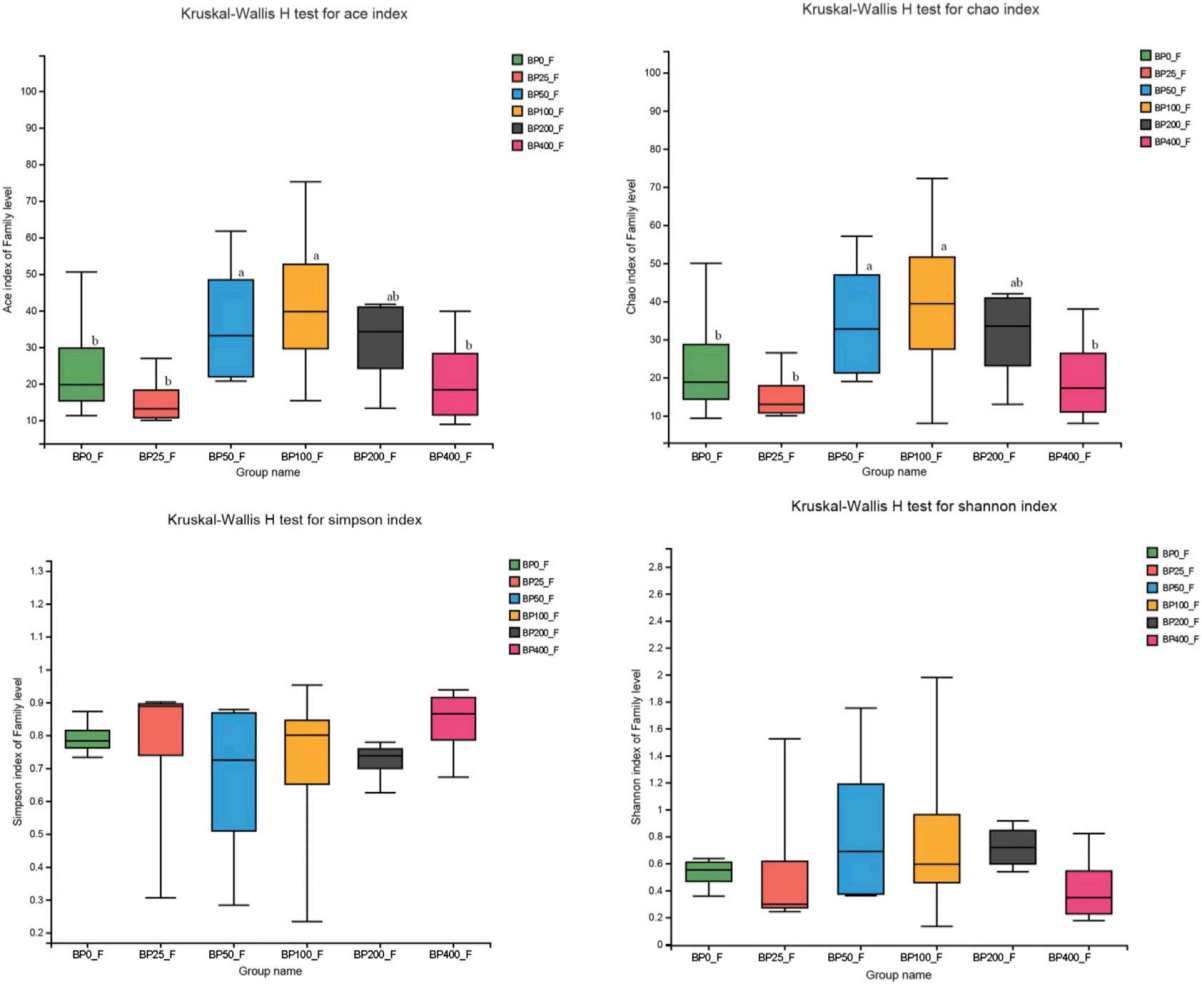
Alpha diversity analysis in foregut.

**FIGURE 3:**
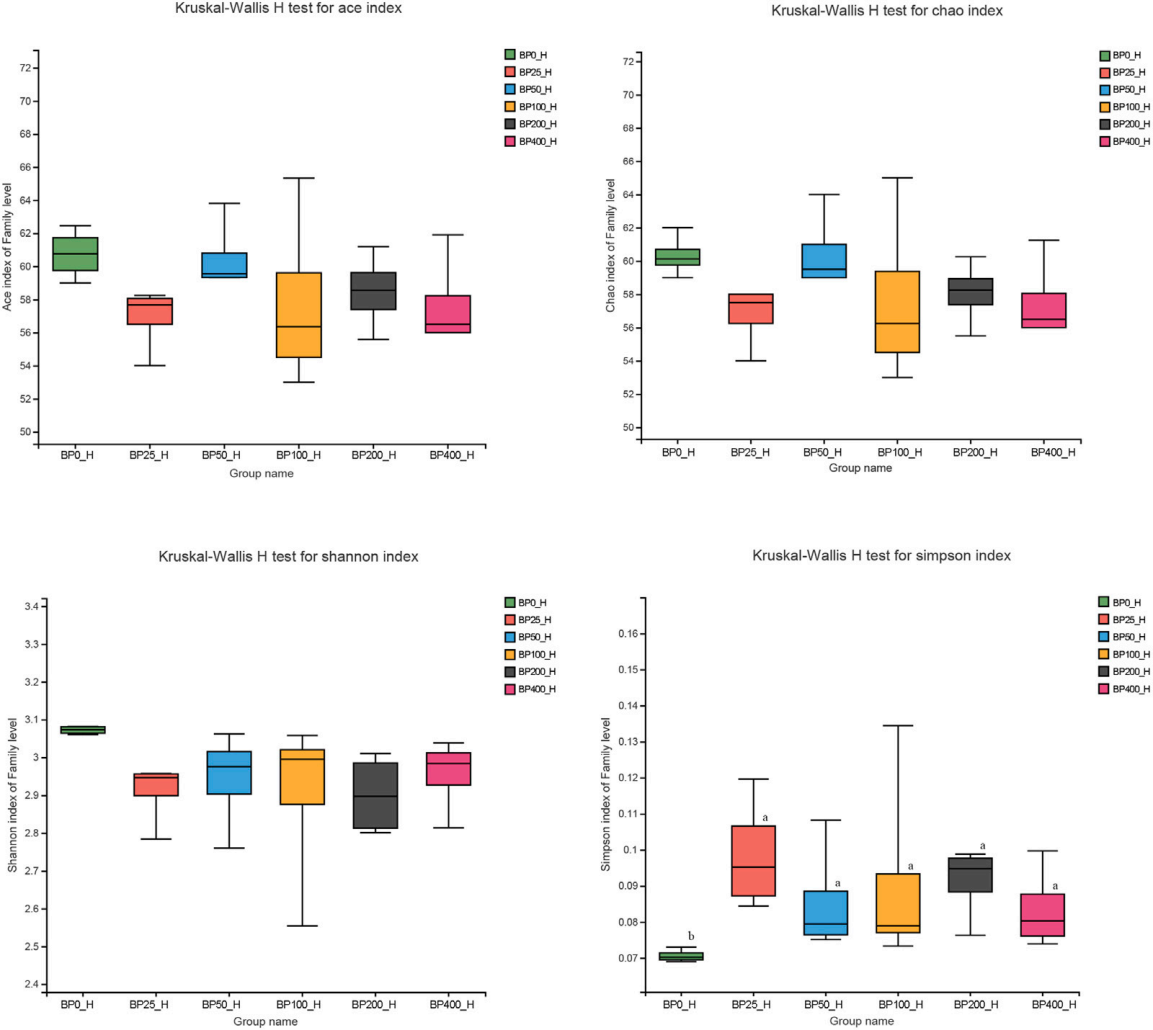
Alpha diversity analysis in hindgut.

**FIGURE 4:**
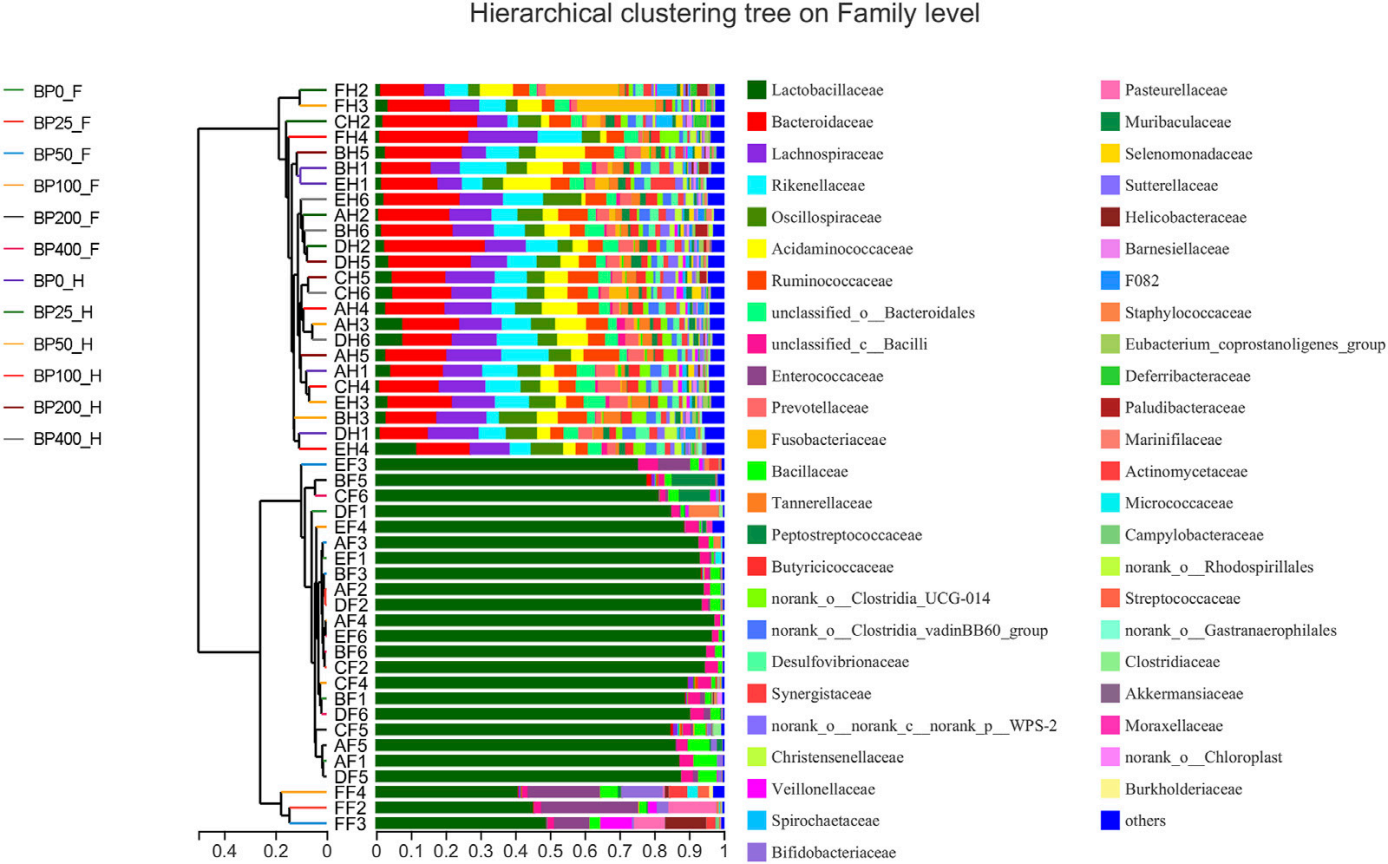
Beta Hierarchical clustering analysis of intestinal microbiota on family level.

**FIGURE 5:**
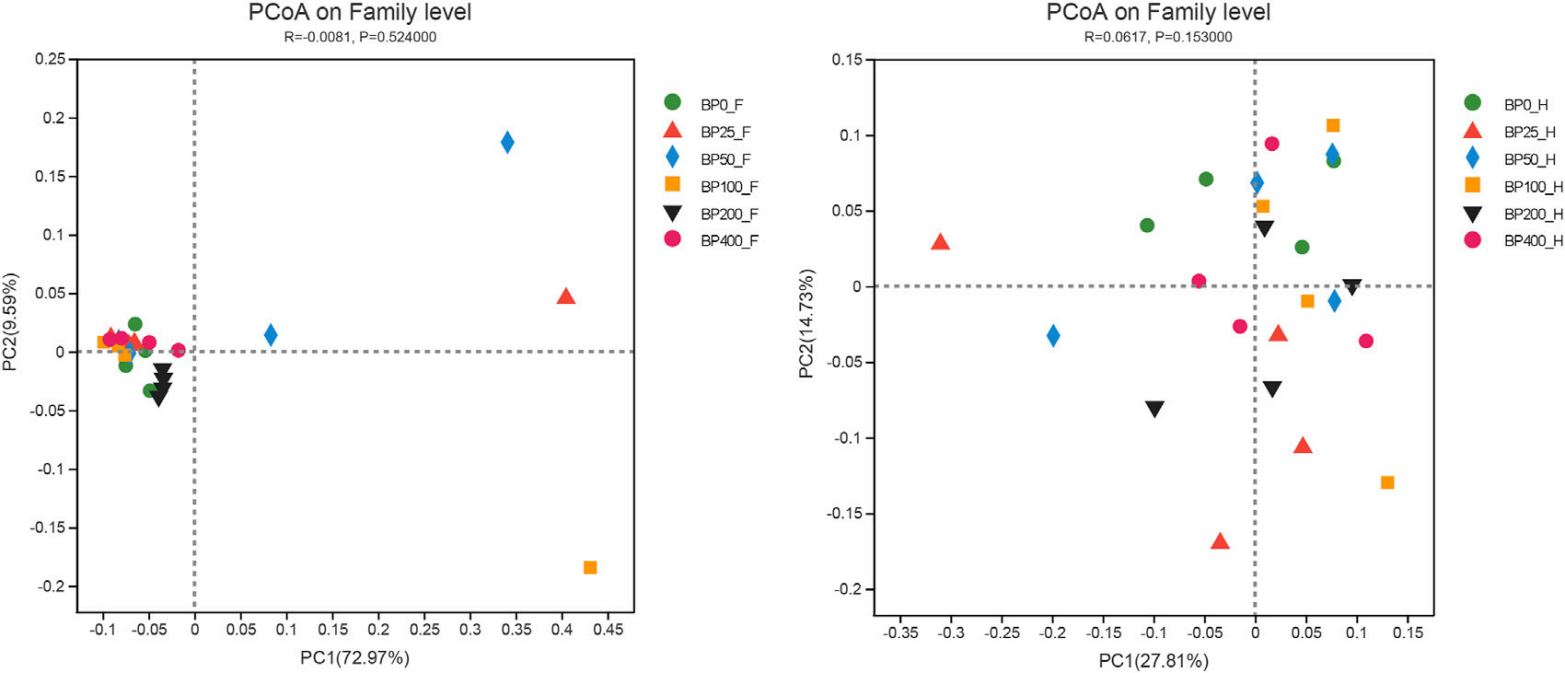
PcoA analysis of intestinal microbiota on family level.

**FIGURE 6:**
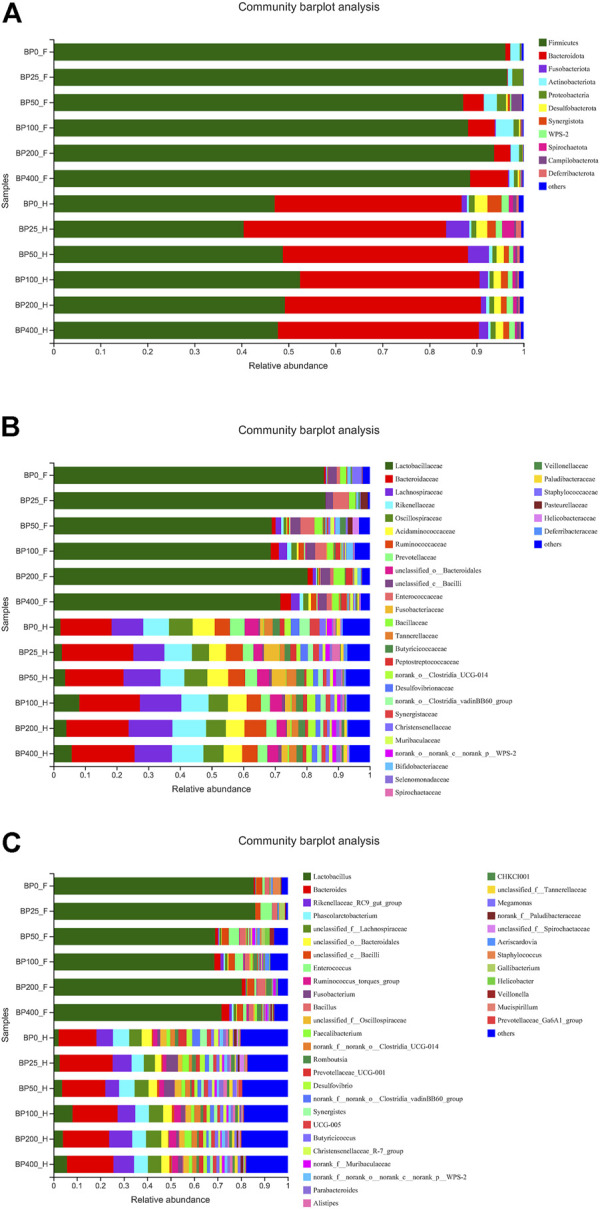
**(A)** The community barplot analysis of intestinal microbiota at phylum level. **(B)** The community barplot analysis of intestinal microbiota at family level. **(C)** The community barplot analysis of intestinal microbiota at genus level.

**FIGURE 7:**
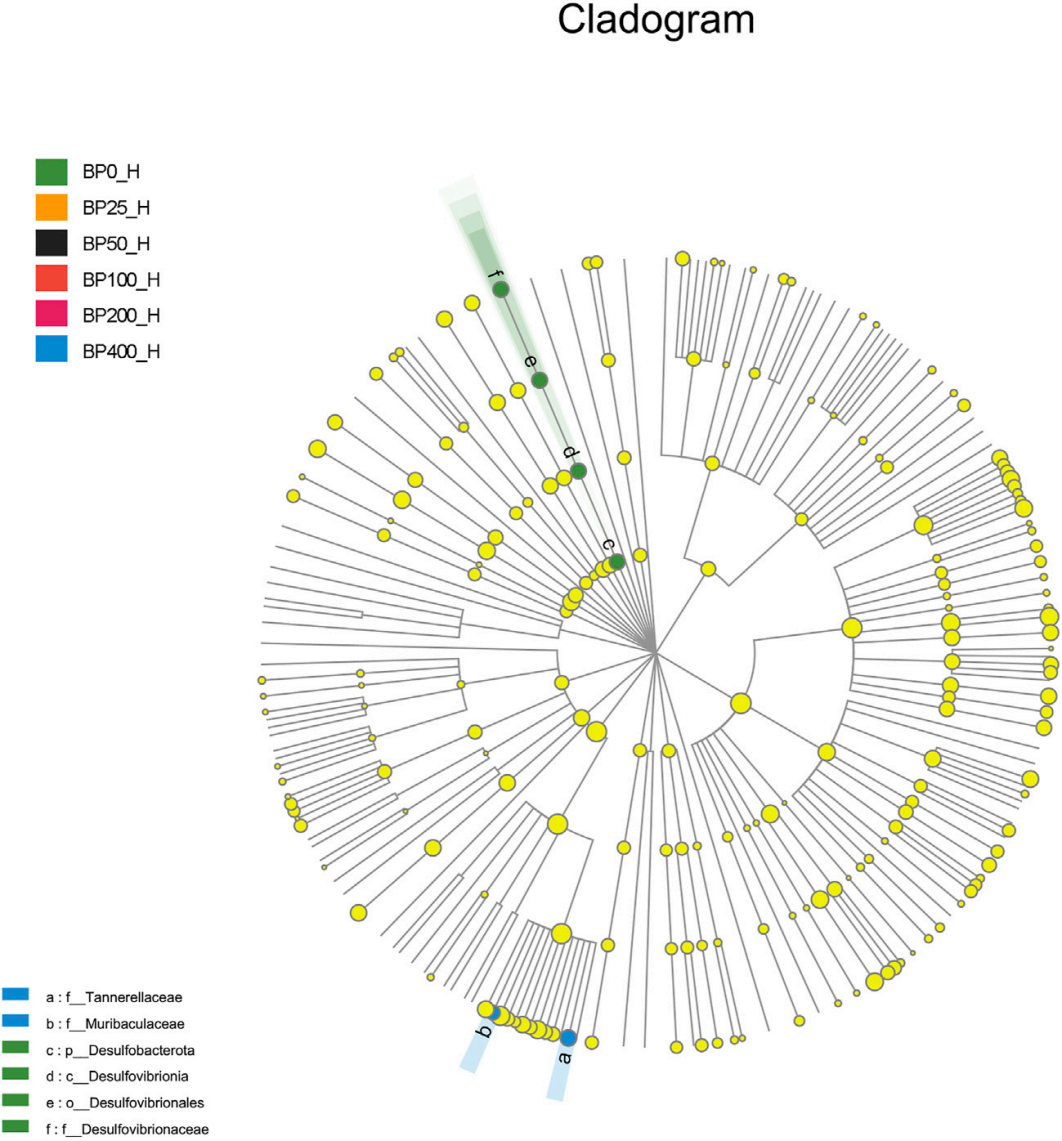
LEfSe analysis of microbiota in hindgut.

A total of 3486992 sequences in the dataset representing 3,657 ASVs were obtained after quality filtering and chimera checking, among which 25 phyla, 178 families, and 376 genera of intestinal microbiota were annotated. As shown in Figure 1A, in the foregut, there were 31, 28, 56, 34, 29, and 28 families and 42, 30, 78, 51, 38, and 34 genera were unique in the BP0, BP25, BP50, BP100, BP200, and BP400 groups, respectively. In the hindgut, there were 29, 29, 28, 29, 31, and 29 families, and 33, 32, 30, 31, 35, and 34 genera were unique in the BP0, BP25, BP50, BP100, BP200, and BP400 groups, respectively (Figure 1B). The Alpha Diversity Analysis showed that the BP50 and BP100 groups exhibited higher diversity of microbiota in the foregut but there was no significant difference among the experimental groups in the hindgut (Figures 2, 3). Hierarchical clustering analysis was performed according to the beta diversity distance matrix, and the UPGMA algorithm was used to construct a tree structure to analyze the degree of difference in the distribution of microbial communities in the anterior and posterior intestines. The results showed that there were significant differences between the foregut microbial communities and hindgut microbial communities (Figure 4). PcoA analysis of the microbial community structure of the foregut and hindgut at the family level demonstrated that Bopu powder affected the microbial community structure in the hindgut but had almost no effect on the microbial community structure of the foregut (Figure 5). Among all experimental groups, the distribution of microflora in the foregut and hindgut was significantly different. At the phylum level, the dominant flora in the foregut is *Firmicutes*, while the dominant flora in the hindgut is *Firmicutes* and *Bacteroidota*, and dietary supplementation with 50–400 mg/kg Bopu powder increased the relative abundance of *Bacteroidota* in the foregut (Figure 6A). At the family level, dietary 25–100 mg/kg Bopu powder supplementation significantly increased the relative abundance of Enterococcaceae. In the hindgut, the highest relative abundance of Bacteroidaceae and Fusobacteriaceae was found in the BP25 group, while the highest relative abundance of Lachnospiraceae was found in the BP100 group (Figure 6B). At the genus level, dietary 25–100 mg/kg Bopu powder supplementation significantly increased the relative abundance of *Enterococcus* in the foregut. In the hindgut, dietary supplementation of 25–50 mg/kg Bopu powder increased the relative abundance of *Bacteroides* and *Fusobacterium*, and dietary supplementation of 50–100 mg/kg Bopu powder increased the relative abundance of *Lactobacillus* (Figure 6C). In addition, the results of LEfSe (Linear discriminant analysis Effect Size) showed that there were significant differences in species of hindgut microbe among the experimental groups. In the hindgut, the relative abundance of Tannerellaceae and Muribaculaceae were significantly enriched in BP50, while BP0 significantly enriched the abundance of Desulfovibrionaceae (Figure 7).

## Discussion

When using new alkaloid resources for animal production, safety is critical. More than 290 components of *Macleaya cordata* have been discovered and/or isolated, with anti-inflammatory, anticarcinogenic, antibacterial, and insecticidal activities (Lin et al., 2018). In animal production, Isoquinoline alkaloids (sanguinarine and chelerythrine) were frequently employed as natural growth promoters with anti-inflammatory and antibacterial properties (Khadem et al., 2014; Ni et al., 2016; Liu et al., 2020). Furthermore, the main constituents of Bopu powder are protopine alkaloids (protopine and allocryptopine), which have anti-inflammatory properties and can be used to treat poultry *E. coli* diarrhea. The oral administration of LD_50_ of protopine in ICR mice was reported to be 313.10 mg/kg, and large dosages of protopine induced brain and liver damage, showing that protopine was moderately hazardous (Hu et al., 2021). In the present study, we found no mortality or morbidity of laying hens, and there was no significant difference in production performances among the experimental groups, indicating that dietary 25–400 mg/kg Bopu powder supplementation had no adverse effects on laying hens. Similarly, a prior study indicated that supplementing broilers with benzophenanthridine and protopine in their drinking water enhanced their productive efficiency index (Previato do Amaral et al., 2016). Furthermore, daily consumption of 1.5 mg of benzophenanthridine alkaloids and protopine alkaloids reduced the unfavorable impacts of extreme heat on feedlot ewes’ growth performance (Estrada-Angulo et al., 2016). This study is the first, to our knowledge, to show that dietary Bopu powder supplementation of 25–400 mg/kg has no deleterious effect on laying hens in a 56-day feeding trial.

The liver function index includes AST and ALT, which were frequently employed as indications of liver health (Diaz et al., 1999; Limdi and Hyde, 2003; Lala and Minter, 2021). Dietary Bopu powder supplementation of 25–400 mg/kg showed no influence on serum AST and ALT activity in this investigation, showing that Bopu powder supplementation had no detrimental effects on the liver of laying hens. Furthermore, serum CHO was linked to liver lipid metabolism, which was significantly elevated in fatty liver-laying hens (Harms and Simpson, 1979; Dong and Tong, 2019; Lin et al., 2021). According to the findings of this study, dietary Bopu powder supplementation significantly reduced serum CHO concentrations, suggesting that Bopu powder supplementation may improve hepatic lipid metabolism and protect the liver against fatty liver disease. Previous research found that protopine extracted from *Fumariaindica Pugsley* has the same hepatoprotective effect as the conventional medication, silymarine (Rathi et al., 2008). Similarly, nutritional supplementation with the antioxidant of plant spices such as garlic and tamarind reduced serum cholesterol in laying hens (Chowdhury et al., 2002; Chowdhury et al., 2005). Furthermore, the liver produces globulin fraction, which contains hundreds of serum proteins, carrier proteins, enzymes, complements, and immunoglobulins, except immunoglobulins, which are produced by plasma cells. In this study, dietary Bopu powder supplementation significantly elevated serum GLB and ALB concentrations, suggesting that Bopu powder could improve liver health and immunological function. It could be because protopine, which contains anti-inflammatory and antioxidant properties, can regulate hepatic lipid metabolism and increase immune cell activation. A study *in vitro* demonstrated that protopine reduced the inflammatory response in lipopolysaccharide-stimulated murine macrophages (Bae et al., 2012). Furthermore, in a carrageenan-induced mouse model, protopine attenuated inflammatory symptoms through modulation of MAPKs/NF-κB signaling cascades (Alam et al., 2019). In this study, dietary Bopu powder supplementation significantly enhanced the serum GSH-Px and catalase activities, demonstrating that Bopu powder increased the antioxidant capacity of laying hens. In line with a previous study, pretreatment of protopine increased serum superoxide dismutase activity in the middle cerebral artery occlusion in rats (Xiao et al., 2007). Similarly, Xiao et al. (2008) demonstrated that protopine relieved H_2_O_2_-induced oxidative stress and apoptosis in PC12 cells. Therefore, supplementation of Bopu powder might ameliorate hepatic lipid metabolism and protect the laying hens from fatty liver disease via antioxidant and anti-inflammatory mechanisms.

Egg quality, including shell and interior quality, is critical to the global egg industry. The hen’s egg is made up of the yolk (30%–33%), albumen (about 60%), and the shell (9%–12%). The Haugh Units were calculated using the thickness of the albumen and the weight of an egg (Haugh, 1937). This was a key indicator of egg albumen quality and related to shelf life. In the present study, dietary 25–50 mg/kg Bopu powder supplementation significantly increased egg Haugh Units. We speculated that it might be attributed to the antioxidant activity of protopine. Meanwhile, similar results were found in a previous study, which demonstrated that dietary antioxidant tea polyphenol supplementation increased the egg HU in hens during the late laying period (Wang et al., 2018). The eggshell contained mainly calcium carbonate, which was formed in the shell gland pouch for more than 15 h. In this study, dietary Bopu powder increased eggshell thickness considerably. Propine administration may have improved the health of the gut and shell gland pouch through anti-inflammatory and antioxidant processes. Similarly, Guo et al. (2021) also demonstrated that dietary *Macleaya cordata* extract (consisting of 7.5% sanguinarine) significantly increased eggshell thickness in Xuefeng black-bone chicken. Hen eggs are healthy foods with balanced nutrition, which contain high-quality proteins and lipids, trace elements, and vitamins. Thus, eggs are easily oxidized by a series of oxidative reactions during storage, resulting in a negative effect on egg nutritional values. In this study, dietary Bopu powder enhanced the antioxidant capacity of the yolk, hence improving egg quality and shelf life. We also discovered that serum antioxidant capacity was positively correlated with yolk antioxidant capacity, implying that Bopu powder supplementation could minimize oxidative stress by regulating laying hen metabolism. A recent study found that dietary antioxidants like selenium-enriched yeast or natural astaxanthin improved the antioxidant capacity of the yolk (Gao et al., 2020; Lin et al., 2020). As a result, nutritional supplementation with Bopu powder may increase egg quality by enhancing laying hen antioxidant capacity.

The gut microbiome has been identified as one of the primary elements influencing laying hen productivity and health. Previous research has linked greater gut microbiota richness and diversity to improved health and productivity (Stanley et al., 2012; Yan et al., 2017). The current study found that dietary Bopu powder supplementation improved the diversity of microbiota in the foregut, implying that supplemented Bopu power may improve the health and productivity of laying hens. Similarly, we discovered that 50 mg/kg Bopu powder administration increased the diversity of microbiota in the foregut, which was consistent with increased serum antioxidant capacity and egg quality in laying hens. We also discovered that the distribution of microbial communities differed significantly between the foregut and hindgut in this investigation. Xiao et al. (2021) discovered that the dominating bacteria in the duodenum were *Lactobacillus*, however, the dominant microorganisms in the cecum and colorectum were more complex, primarily comprising *Bacteroides*, *Odoribacter*, and *Clostridiales vadin BB60* group in laying hens. At the phylum level, *Firmicutes* was the main flora in the foregut, while *Firmicutes* and *Bacteroidota* were the dominant flora in the hindgut in this study. The addition of 50–400 mg/kg of Bopu powder to the diet increased the relative abundance of *Bacteroidota* in the foregut. Interestingly, *Bacteroidota* is commonly seen as a “generalist” degrader of dietary fiber, implying that supplementing with Bopu powder may boost fiber fermentation and short-chain fatty acid (SCFA) production. At genus level, dietary 25–100 mg/kg Bopu powder supplementation enriched *Enterococcus* in the foregut, Bacteroidaceae, Fusobacteriaceae, and Lachnospiraceae in the hindgut. *Enterococcus* was assumed to be a natural antibacterial probiotic capable of preventing diarrhea, improving feed efficiency, and promoting animal production growth (Franz et al., 2011). Bacteroidaceae was considered as a probiotic for its ability to degrade complex polysaccharides and produce acetate, propionate, or succinate (Polansky et al., 2016; Medvecky et al., 2018). Fusobacteriaceae could modulate the growth of other bacterial species through metabolic by-products, which was helpful for the maintenance of an overall microbial structure. Similarly, Wang *et al.* (2020) found that dietary probiotic *Bacillus subtilis* supplementation increased the abundances of *Fusobacteria* phylum, *Fusobacteriia* class, Fusobacteriaceae family, and *Fusobacterium* genus and improved the performance of breeding geese during the laying period. Lachnospiraceae was involved in the production of SCFAs (butyrate, propionate, and acetate), which were important energy sources for intestinal epithelial cells (Rychlik, 2020). In recent studies, Lachnospiraceae have been shown to be enriched in long-living Italian and Chinese populations (Kong et al., 2016). Given that egg layers have a 1-year lifespan of production, enrichment with Lachnospiraceae could have a significant favorable impact on health and productivity. In addition, LEfSe analysis showed that the relative abundance of Tannerellaceae and Muribaculaceae were significantly enriched in the BP50 group, while the BP0 group significantly enriched the abundance of Desulfovibrionaceae in the hindgut. Previous studies found that the Tannerellaceae were enriched in certain gastrointestinal disorders, such as Crohn’s disease, indicating that Tannerellaceae might be related to intestinal immunity (Hernández et al., 2019). Furthermore, Zhao et al. (2020) demonstrated that Tannerellaceae was negatively correlated with immune traits, whereas Muribaculaceae was positively correlated with immune traits. In the present study, Tannerellaceae and Muribaculaceae were both enriched in hindgut in the BP50 group and positively correlated with improving egg quality and antioxidant capacity of laying hens. Thus, we speculated that the enrichment abundance of Tannerellaceae and *Muribaculacea*e might improve the gut health of laying hens. Chang et al. (2022) discovered that orange corn (high carotenoids) diet significantly enriched Tannerellaceae in laying hens compared to white corn (low carotenoids) diet. Desulfovibrionaceae could release sulfate in the gut and produce hydrogen sulfide. A larger concentration of hydrogen sulfide, on the other hand, was hazardous and could cause inflammatory bowel disease in the colon (Guo et al., 2016). In laying hens with an average age of 64 weeks (Joat et al., 2021), the abundance of Desulfovibrionaceae increased significantly, showing that Desulfovibrionaceae was negatively correlated with intestinal health.

Surprisingly, dietary low-dose 25–50 mg/kg Bopu powder supplementation had a greater impact on egg quality and antioxidant status in laying hens but high-dose, 100–400 mg/kg, groups had no additive effects. The effects of Bopu powder supplementation were connected with the diversity of gut microbiota in the current investigation. It is possible that low-dose Bopu powder supplementation improved egg quality and antioxidant capacity of layers by increasing the diversity of gut microbiota and the abundance of beneficial bacteria, whereas high doses reduced the diversity of gut microbiota due to Bopu powder’s antibacterial effect. Furthermore, excessive Bopu powder supplementation may raise drug metabolism stress in laying hens, thereby counteracting the synergistic effects of Bopu powder.

## Conclusion

In the 56-day trial, dietary Bopu power supplementation of 25–400 mg/kg had no negative effects on laying hens. Furthermore, supplementing laying hens with 50 mg/kg Bopu powder improved egg quality and antioxidant capacity, which could be attributed to an increase in intestinal microbiota richness and changes in microbial composition, particularly the enrichment of *Enterococcus* in the foregut and *Bacteroidaceae*, *Fusobacteriaceae*, and *Lachnospiraceae* in the hindgut. Thus, Bopu powder may be utilized in laying hens to improve egg quality and intestinal health, and we proposed that the ideal dietary dose of Bopu powder in laying hens was 25–50 mg/kg.

## Data Availability

The original contributions presented in the study are included in the article/Supplementary Material, further inquiries can be directed to the corresponding authors.
